# Diagnostic Challenge: The Case of a 70‐Year‐Old Man Who Presents With Progressive Vision Loss and Headaches

**DOI:** 10.1002/acn3.70089

**Published:** 2025-07-25

**Authors:** Malya Sahu, Rohini Samudralwar

**Affiliations:** ^1^ Hospital of the University of Pennsylvania Philadelphia Pennsylvania USA

**Keywords:** headaches, leptomeningeal carcinomatosis, optic neuropathy

## Abstract

A 70‐year‐old presents with 6 weeks of progressive bilateral vision loss and headaches. He was also recently treated for presumed sciatica. His exam on admission is notable for poor visual acuity (no light perception), optic nerve atrophy bilaterally with swelling and disc hemorrhage on the left eye and left foot drop. MRI orbits reveals diffusion restriction and enhancement of bilateral optic nerves extending into the optic chiasm. He undergoes bilateral temporal artery biopsy due to concern for giant cell arteritis and high‐dose steroids with no improvement in vision and both biopsies are normal. Ultimately, full neuro‐axis imaging reveals evidence of leptomeningeal enhancement of the cerebellum, cervical and thoracic spine, and cauda equina nerve roots, as well as bilateral posterior semicircular canal enhancement and developing hydrocephalus. A CNS diagnosis is made via lumbar puncture, though ultimately the patient eventually expires in the hospital.

## 1 Summary of Case (HPI, Relevant Exam Findings, and Relevant Data)

**FIGURE 1:**
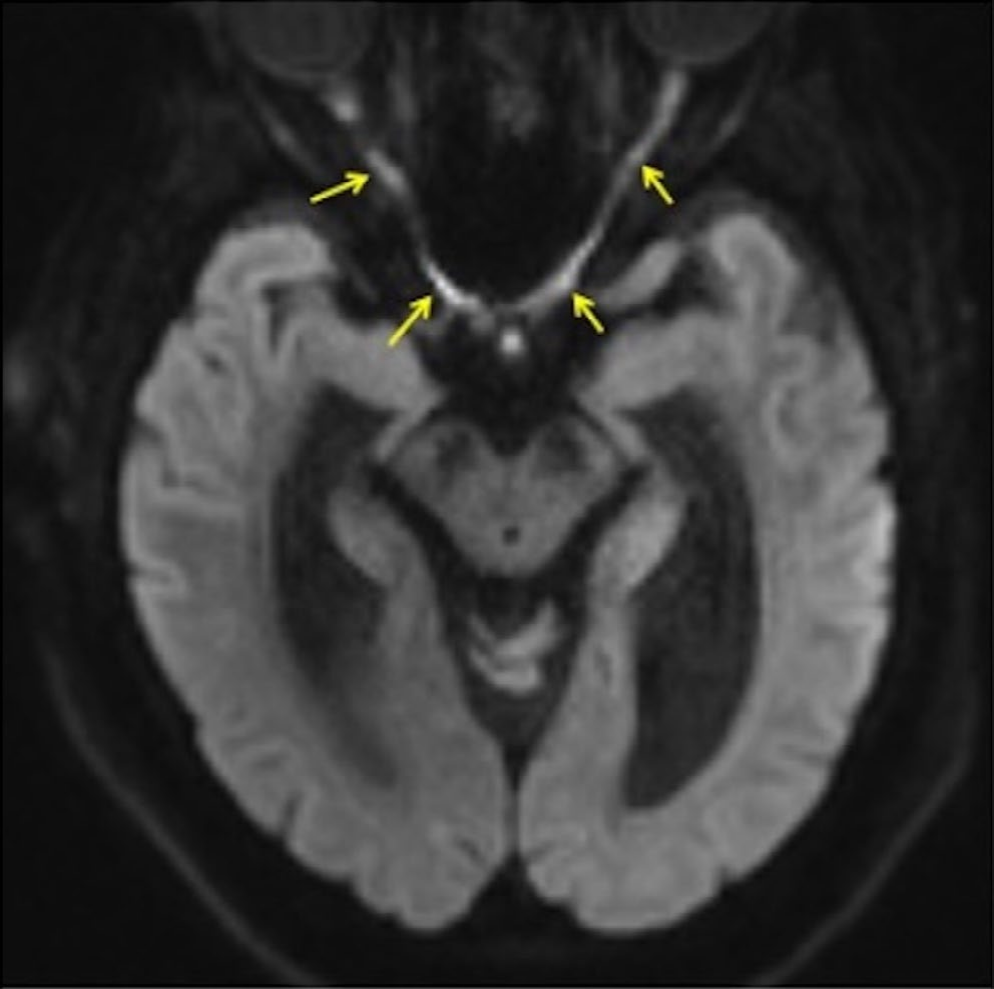
Restricted diffusion in bilateral optic nerves and chiasm.

**FIGURE 2:**
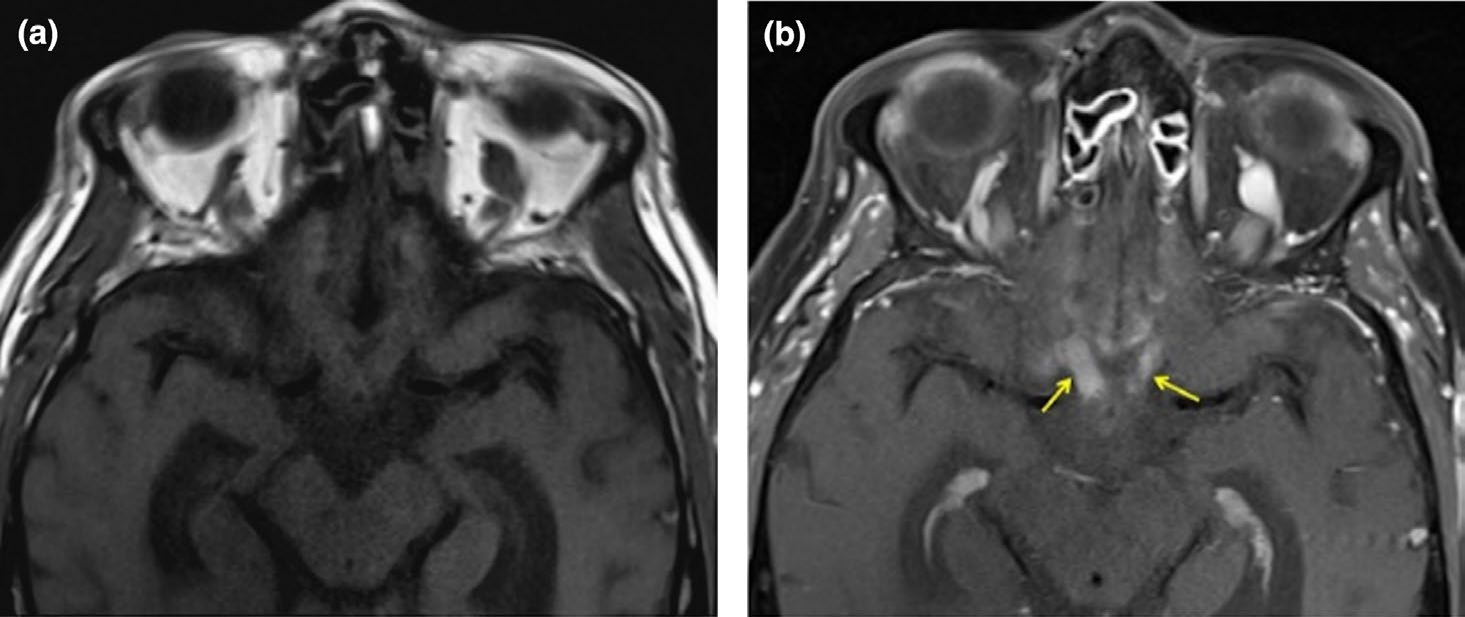
Enhancement and enlargement of the pre‐chiasmatic intracranial optic nerves and chiasm, right greater than left, in pre‐ (a) and post‐contrast (b) T1 images.

**FIGURE 3:**
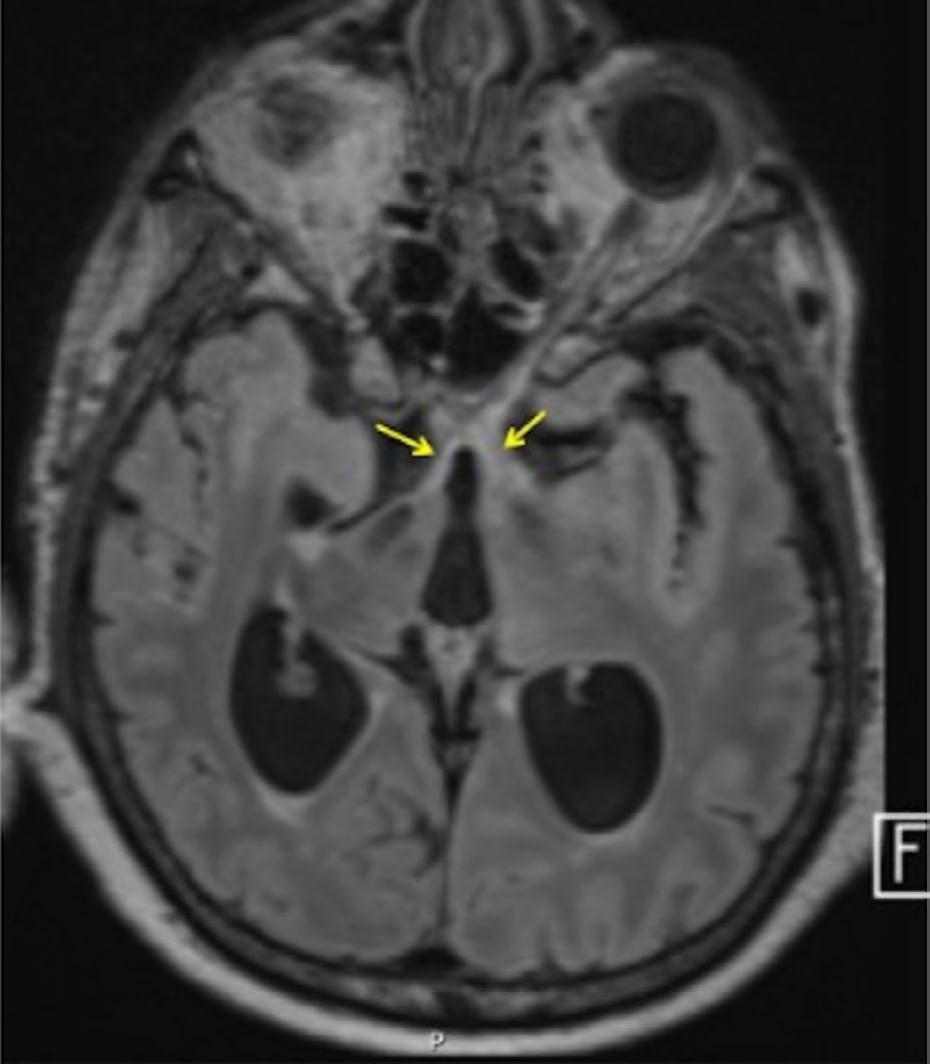
T2/FLAIR: Extension of FLAIR signal abnormality into the post‐chiasmatic optic tracts.

**FIGURE 4:**
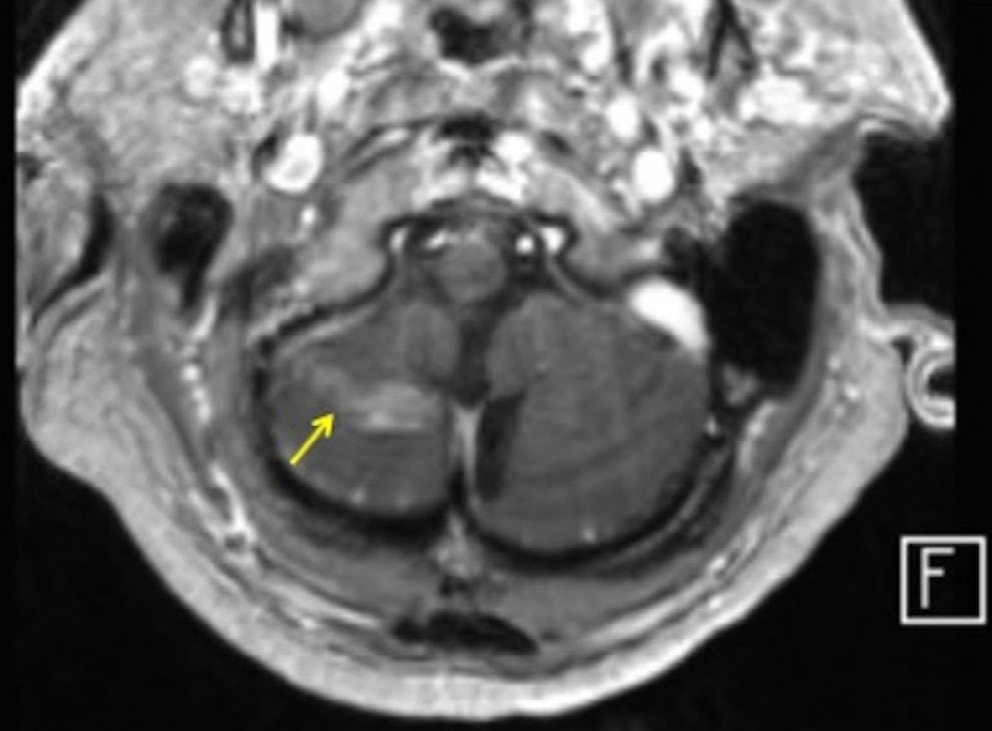
T1 post‐contrast: Right greater than left cerebellar leptomeningeal enhancement.

**FIGURE 5:**
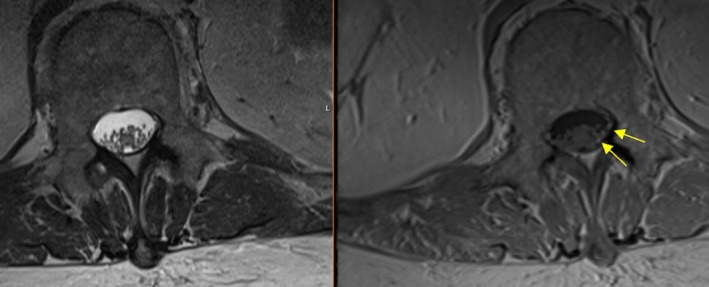
T2 (left) and T1 post‐contrast (right): Leptomeningeal enhancement around the cauda equina nerve roots. Arrows are pointing to enhancing cauda equina nerve roots as they traverse the thecal sac at/below the level of the conus.

A 70‐year‐old presents with 6 weeks of progressive bilateral vision loss and headaches. He was also recently treated for presumed sciatica. His exam on admission is notable for poor visual acuity (no light perception), optic nerve atrophy bilaterally with swelling and disc hemorrhage in the left eye and left foot drop. MRI orbits reveal diffusion restriction and enhancement of bilateral optic nerves extending into the optic chiasm (Figures 1 and 2). He undergoes bilateral temporal artery biopsy due to concern for giant cell arteritis and high‐dose steroids with no improvement in vision, and both biopsies are normal. Ultimately, full neuro‐axis imaging reveals evidence of leptomeningeal enhancement of the cerebellum, cervical and thoracic spine, and cauda equina nerve roots, as well as bilateral posterior semicircular canal enhancement and developing hydrocephalus (Figures 3, 4, 5). Lumbar puncture was notable for elevated opening pressure, markedly elevated CSF protein, and ultimately, CSF cytopathology revealed adenocarcinoma. PET imaging revealed increased uptake in the upper gastric region, which was biopsied once with no malignant cells found. Ultimately, the patient and family elected to transition to hospice prior to identification of a primary malignancy, and autopsy was not pursued.

## 2 Diagnosis

Leptomeningeal carcinomatosis secondary to adenocarcinoma of either gastric or bladder origin.

## 3 Take‐Home Points

- Leptomeningeal carcinomatosis may present clinically as sudden, painless vision loss due to an ischemic/inflammatory neuropathy, which is challenging to diagnose and requires a high degree of clinical suspicion [1].
- MRI diffusion restriction, enhancement, and FLAIR signal changes of bilateral optic nerves extending into the chiasm support the need to consider *early neuroaxis imaging* and *lumbar puncture* to avoid misdiagnosis, unnecessary empiric treatment, and delays in appropriate management.
- The concurrent development of other neurologic symptoms (i.e., left foot drop as in our patient) with sudden vision loss warrants an evaluation of systemic etiologies, rather than primary ocular.
- Leptomeningeal carcinomatosis due to an adenocarcinoma does not necessarily require systemic lymphadenopathy.
- While biopsy‐confirmation of the primary site of malignancy was not achieved in this case, leptomeningeal carcinomatosis may be due to uncommon tumors (such as gastric or bladder) and should be on the differential for new vision or neurologic symptoms in a patient with history of any malignancy [2, 3, 4].

## Author Contributions

The author takes full responsibility for this article.

## Conflicts of Interest

The authors declare no conflicts of interest.

## Data Availability

The authors have nothing to report.
